# 非小细胞肺癌肺部寡转移的手术和热消融治疗现状与展望

**DOI:** 10.3779/j.issn.1009-3419.2023.106.06

**Published:** 2023-03-20

**Authors:** Baodong LIU

**Affiliations:** 100053 北京，首都医科大学宣武医院胸外科; Department of Thoracic Surgery, Xuanwu Hospital, Capital Medical University, Beijing 100053, China

**Keywords:** 肺肿瘤, 寡转移, 局部治疗, 手术, 热消融, Lung neoplasms, Oligometastases, Local therapy, Surgery, Thermal ablation

## Abstract

寡转移可视为早期转移和广泛转移之间的过渡状态（肿瘤负荷有限、独特的肿瘤生物学行为），由于转移灶的数量和受累器官的数量相对有限，经过积极的系统治疗和局部治疗，有潜在治愈的机会。随着分子靶向药物治疗和免疫治疗的飞速发展，寡转移性非小细胞肺癌（non-small cell lung cancer, NSCLC）包括寡复发和寡进展病灶的局部治疗越来越受到重视。本文对手术和热消融局部治疗手段的相关研究、疗效及影响因素、安全性和适应证等进行了探讨。

## Abstract (en)

Oligometastasis can be regarded as a transition state between early metastasis and extensive metastasis (limited tumor load, unique tumor biological behavior). Due to the relatively limited number of metastatic foci and the number of affected organs, there is a potential chance of cure after active systemic and local treatment. With the rapid development of molecular targeted drug therapy and immunotherapy, local therapy for oligometastatic non-small cell lung cancer (NSCLC), including oligorecurrence and oligoprogression, has received increasing attention. In this paper, the relevant research, efficacy, influencing factors, safety and indications of local therapy such as surgery and thermal ablation were discussed.

## 1 背景

1975年Martini和Melamed^[1]^发表了50例多原发肺癌的治疗结果，发现多原发肺癌并不是手术的排除标准，其总生存与单发肺癌相似，这一结果引发了临床医生对肺癌寡转移灶局部治疗的兴趣。

临床上，非小细胞肺癌（non-small cell lung cancer, NSCLC）占全部肺癌的85%-90%，患者在诊断时早期（I期-II期）占17%，局部中晚期（III期）占22%，晚期占57%^[2]^。I期-IIIA期患者肺叶切除+纵隔淋巴结清扫术后有30%-55%会出现局部复发和远处转移^[3]^。然而，并非所有的远处转移都是不可治愈的，在第八版肺癌肿瘤原发灶-淋巴结-转移（tumor-node-metastasis, TNM）分期系统中，同一肺叶内存在其他肿瘤结节（T_3_）术后5年生存率约为30%（60%合并N_1_/N_2_），同侧不同肺叶内肿瘤结节（T_4_）术后5年生存率约为13%（60%合并N_1_/N_2_），对侧肺叶肿瘤结节（M_1a_）术后没有5年生存率，肺外存在单发转移结节（M_1b_）的前瞻性研究中位生存期约为11个月^[4,5]^。

## 2 基本概念和分类

1995年Hellman和Weichselbaum^[6]^共同提出了“寡转移（oligometastases）”的概念，用以描述数量及分布有限的远处转移性疾病状态。2011年，Weichselbaum和Hellman^[7]^对这一概念又进行了更新。Niibe等^[8]^随后又提出了“寡复发”的概念。

2020年Guckenberger等^[9]^代表欧洲放射治疗和肿瘤学会（European Society for Radiotherapy, ESTRO）和欧洲癌症研究治疗组织（European Organisation for Research and Treatment of Cancer, EORTC）使用第二轮德尔菲法将寡转移性疾病的17个特征归于四类，联合提出了寡转移分类共识。为便于临床应用，结合既往文献意见，作者建议对寡转移的概念和分类简化如下：（1）寡转移一般指同时性或异时性寡转移，包括诊断原发性肺肿瘤的同时诊断的寡转移，或在原发性肺肿瘤系统治疗后不久诊断的寡转移；（2）寡复发是指原发性肺肿瘤局部治疗后出现的局部或肺内其他部位的复发；（3）寡进展是指患者经过系统治疗后残留病灶有限或只有一个病灶进展（图1）。

**图1:**
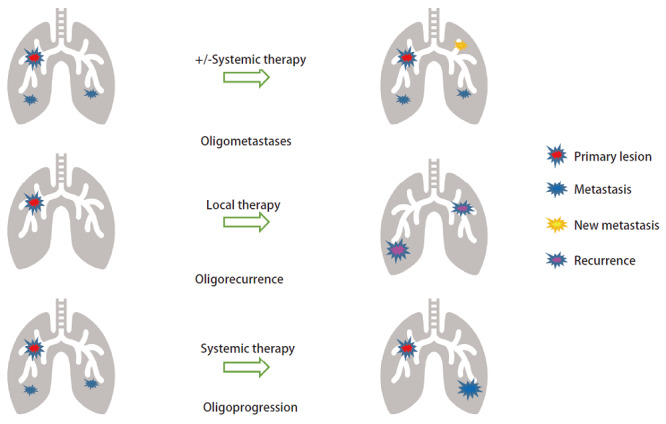
肺癌肺部寡转移分类示意图

在一项meta分析^[10]^中，大多数NSCLC患者的寡转移部位位于脑或肺部，其次是肾上腺、骨头、肝脏和淋巴结等。欧洲临床医生定义了寡转移性肺癌最多为三个器官的五个病变^[11]^。这一标准被泛欧洲多学科共识小组所推荐^[12]^，该共识不认为纵隔淋巴结转移属于寡转移站点；并建议强制性的正电子发射计算机断层扫描（positron emission tomography/computed tomography, PET/CT）和头颅核磁共振（magnetic resonance imaging, MRI）分期；还建议对孤立性转移病灶活检，除非多学科团队（multidisciplinary team, MDT）认为风险较大。

## 3 治疗现状

寡转移可视为早期转移和广泛转移之间的过渡状态（肿瘤负荷有限、独特的肿瘤生物学行为），由于转移灶的数量和受累器官的数量相对有限，经过更积极的局部治疗，有潜在治愈的机会。但是目前国内对寡转移认识不足，在治疗上多以系统治疗为主，尤其是在过去十年里，分子靶向药物治疗和免疫治疗取得了令人瞩目的发展。然而随着胸腔镜手术和热消融技术的广泛开展，对于局部治疗后的寡复发、系统治疗后的寡进展等寡转移性NSCLC患者采用局部巩固治疗可以改善总生存和无进展生存期（progression-free survival, PFS）。局部治疗或局部巩固治疗包括手术、放疗和热消融等，其疗效主要取决于MDT管理模式。

### 3.1 局部治疗

局部治疗或局部巩固治疗在英文文献中有以下表达方式：LCT（local consolidative therapy）、LAT（locally-aggressive therapy）、ATT（aggressive thoracic therapy）、RCT（radical consolidative treatment）和RLT（radical local treatment）等。

#### 3.1.1 寡复发

Torok等^[13]^回顾性分析了1995年-2009年1,719例I期-III期NSCLC术后患者，368例（21%）有远处转移，115例患者（31%）单发转移，69例（19%）患者存在2个-3个转移灶（其中50%为寡转移），采用LAT治疗。中位随访时间为39个月，寡转移和广泛转移的中位生存期分别为12.4个月和6.1个月[风险比（hazardratio, HR）=0.54，95%CI：0.42-0.68，P<0.001]；单发转移患者的中位生存期最长为14.7个月；年轻、寡转移、化疗是预后良好的因素。

#### 3.1.2 寡转移

Kwint等^[14]^回顾性分析了2008年-2016年91例IV期NSCLC患者经过系统治疗+RLT。中位随访期为35个月，中位PFS为14个月（2个月-89个月，95%CI：12个月-16个月），中位生存期为32个月（3个月-89个月，95%CI：25个月-39个月）；1年生存率为85%，2年生存率为58%；1年PFS为55%，2年PFS为27%。Xu等^[15]^回顾性分析了2010年10月-2016年5月145例IV期表皮生长因子受体（epidermal growth factor receptor, EGFR）（+）NSCLC患者（诊断2个月内，≤5个肿瘤）一线EGFR酪氨酸激酶抑制剂（tyrosine kinase inhibitors, TKIs）治疗后LAT治疗的结果：其中51例（35.2%）的全部寡转移灶接受LAT治疗（全部LAT组），55例（37.9%）在原发灶或寡转移灶接受LAT治疗（部分LAT组），39例（26.9%）未接受任何LAT治疗（非LAT组）。全部LAT组、部分LAT组和非LAT组的中位PFS分别为20.6个月、15.6个月和13.9个月（P<0.001）；中位生存期分别为40.9个月、34.1个月和30.8个月（P<0.001）。中位生存期明显提高，原发肿瘤（40.5个月 vs 31.5个月，P<0.001）、脑转移瘤（38.2个月 vs 29.2个月，P=0.002）和肾上腺转移瘤（37.1个月 vs 29.2个月，P=0.032）。放疗3级以上不良事件包括肺炎（7.7%）和食管炎（16.9%）。Mitchell等^[16]^回顾性分析了2000年-2017年88例cT_1-3_N_0-2_M_1_期NSCLC患者（同时性转移≤3个）进行LCT治疗的结果。63例（71.6%）接受放疗，25例（28.4%）接受手术治疗，包括肺叶切除20例（80.0%）、全肺切除3例（12.0%）和亚肺叶切除2例（8.0%）。90天死亡率：手术为0%（0/25），放疗为1.6%（1/63）；中位生存期：手术为55.2个月，放疗为23.4个月；1年生存率分别为95.7%和74.3%，5年生存率分别为48.0%和24.2%；倾向匹配评分提示手术生存优于放疗。Antonoff等^[17]^回顾性分析了2000年-2017年194例cT_1-3_N_0-2_M_1_期NSCLC患者（原发肿瘤切除，同时性转移≤3个）进行LCT治疗的结果。173例（89%）接受LCT，30例（15%）原发肿瘤切除，25例（83%）开胸手术；肺叶切除25例（83%），全肺切除3例（10%），亚肺叶切除2例（7%）；平均失血量为200 mL（50 mL-600 mL），手术时间为200 min（72 min-492 min）；4例（15%）需要控制近端肺动脉，4例（15%）需要袖式切除术，2例患者（7%）需要计划外手术，3例患者（11%）行胸壁切除术，9例（33%）有门钉淋巴结，16例（59%）手术难度增加，提示手术存在一定的难度和风险。

2016年报道了一项前瞻多中心双臂随机II期临床研究NCT01725165（系统治疗 vs 系统治疗+LCT）^[18]^，入组标准为组织学证实为IV期NSCLC：一线化疗4个及以上周期或者EGFR或者间变性淋巴瘤激酶（anaplastic lymphoma kinase, ALK）-TKIs治疗3个月及以上，然后1:1随机分组，LCT治疗（放疗或手术，n=25，其中24%的患者接受了手术）和维持治疗（n=24）。中位随访12.39个月，PFS在LCT治疗组为11.9个月，维持治疗组为3.9个月；1年PFS分别为48%和20%；中位生存期显示获益（42.1个月 vs 17.0个月）。不良事件类似（无4级以上）。研究于2019年再次报道研究结果^[19]^，中位随访38.8个月，PFS在LCT治疗组为14.2个月，维持治疗组为4.4个月；中位生存期分别为37.6个月和9.4个月。

Uhlig等^[20]^利用美国国家癌症数据库（National Cancer Database, NCDB）分析了2010年-2015年34,887例患者，手术+系统治疗为835例，外照射或热消融+系统治疗为9,539例，系统治疗为24,513例。多因素分析发现，手术切除 vs 系统治疗的HR为0.59（95%CI: 0.55-0.64, P<0.001），手术切除 vs 外照射/热消融的HR为0.62（95%CI: 0.57-0.67, P<0.001），外照射/热消融 vs 系统治疗的HR为0.95（95%CI: 0.93-0.98, P=0.002）。外照射/热消融生存获益在T_1-2 _N_0-1_伴有单发转移的IV期鳞癌中尤其明显（HR=0.68, 95%CI: 0.57-0.80, P<0.001），1年生存率为60.4% vs 45.4%，2年生存率为32.6% vs 19.2%，3年生存率为20.2% vs 10.6%。

### 3.2 手术切除

手术切除是治疗肺寡转移的主要方法之一。从安全性和有效性来说，手术再切除存在一定的难度且并发症多，所以需要慎重选择。

#### 3.2.1 寡复发

2002年12月-2011年6月，Endo等^[21]^进行了一项前瞻性多中心研究，研究对象为34例cT_1-2_N_0-1_肺癌伴单器官转移或pT_1-2_N_0-1_肺癌完全切除后出现单器官异时性转移患者。转移灶分为三组：A组为除脑、肺外的单器官转移；B组有同时性脑转移；C组有肺转移。治疗干预措施为手术切除异时性转移或切除原发灶和同时性转移灶。良性病变没有转移为6例（18%），原发性肺癌病灶不完全切除为5例（15%），原发性肺癌原发灶和转移灶完全切除为20例（59%），该20例患者的5年生存率为44.7%。研究认为cT_1-2_N_0-1_期肺癌伴单器官转移是手术切除的良好选择，预期5年生存率约为40%，与II期NSCLC相当。

#### 3.2.2 寡转移

一项meta分析^[22]^对20项研究757例有1个-5个同时或异时性转移NSCLC患者进行分析，训练集和验证集分别为2/3和1/3的患者。手术是原发肿瘤（635例，83.9%）和转移瘤（339例，62.3%）最常用的治疗方法，52.4%只接受手术。中位生存期为26个月，1年生存率为70.2%，5年生存率为29.4%。同时性与异时性（P<0.001）、淋巴结分期（P=0.002）、腺癌（P=0.036）可以预测生存期。风险分组采用递归分区分析（recursive partitioning analysis, RPA）显示：低风险-异时性转移患者的5年生存率为47.8%，中风险-同时性转移合并N_0_疾病患者的5年生存率为36.2%，高风险-同时性转移合并N_1_/N_2_疾病患者的5年生存率为13.8%。

#### 3.2.3 寡进展

Yu等^[23]^回顾性分析了EGFR-TKIs治疗获得性耐药的18例NSCLC患者采用局部巩固治疗：≤5个肿瘤，85%的患者在局部巩固治疗后1个月内重新开始EGFR-TKIs治疗。局部巩固治疗后中位PFS1（局部巩固治疗开始到局部进展）为10个月（95%CI：2个月-27个月），中位时间PFS2（局部巩固治疗开始到系统治疗发生改变）为22个月（95%CI：6个月-30个月）；局部巩固治疗的中位总生存期为41个月（95%CI：26个月-未达到）。

#### 3.2.4 疗效及影响因素

手术治疗寡转移性NSCLC患者的中位生存期为26个月-42.1个月，中位PFS为11.9个月-20.6个月；1年生存率为60%-95%，2年生存率为32.6%-48%，5年生存率为25.1%-48%。文献^[24]^报道，以生存率计算，T_3_患者约为28%、T_4_约为21%，脑转移灶切除为11%-30%，肾上腺转移灶切除约为26%。年轻患者、肿瘤较小、异时性病灶、缺乏淋巴结受累与预后良好相关。T_1-2_、N_0-1_伴有单发转移灶患者的生存时间要比多发转移灶的长。手术不推荐全肺切除和亚肺叶切原发灶。

#### 3.2.5 安全性

肺部寡转移或寡复发灶的手术再切除存在一定的难度且并发症多，应当慎重选择。文献^[25]^报道手术并发症发生率及死亡率一般低于2%。

#### 3.2.6 适应证

术前评估包括肿物能够手术切除，需要除外纵隔淋巴结和远处转移，评价患者的心肺肝肾功能可耐受手术。T_3_期患者没有纵隔或远处转移，建议通过肺叶切除术进行治疗（证据级别1B）；T_4_期患者没有纵隔或远处转移，建议在确保患者有充足肺功能储备的前提下切除每一个病灶（证据级别1B）；M_1a_期患者在确保患者有充足肺功能储备的前提下切除每一个病灶（证据级别2C）；M_1b_期患者有脑或肾上腺寡转移性疾病的可以手术切除^[4]^。

### 3.3 热消融

热消融技术具有微创、患者恢复快、安全、并发症少、适形、效果可靠、操作简单、可以重复进行等优点，可用于无法手术和放疗的患者。

#### 3.3.1 寡复发

Ni等^[26]^回顾性分析了2012年6月-2020年1月103例NSCLC患者术后135个肺寡复发（出现中位时间间隔为术后34.8个月）灶微波消融（microwave ablation, MWA）的结果。中位PFS和生存期分别为15.1个月和40.6个月；1年、3年和5年PFS率分别为64.1%、24.4%和9.2%；1年、3年和5年生存率分别为97.1%、58.7%和34.3%。MWA术后15例（14.6%）患者局部复发，45例（43.7%）患者出现胸内寡复发，20例（19.4%）患者出现远处转移。在多因素分析中，局部复发和胸内复发不是生存期的预测因素（P=0.23, P=0.26）。然而，远处转移可预测生存期（HR=5.37, 95%CI: 1.04-27.84, P=0.04）。Kodama等^[27]^回顾性分析了2003年5月-2010年10月44例I期-IV期术后NSCLC患者（同侧复发63.6%，对侧复发36.4%）的射频消融（radiofrequency ablation, RFA）结果，患者有手术禁忌、≤5个肿瘤、无肺外转移，肿瘤平均直径为17 mm（6 mm-40 mm）。中位随访为28.6个月（1个月-98个月），1年生存率为97.7%，3年生存率为72.9%，5年生存率为55.7%；1年和3年的肿瘤无复发生存率分别为76.7%和41.1%。肿瘤大小和性别是多因素分析中重要的独立预后因素，其中18例女性的5年生存率为73.3%，38例≤3 cm的小肿瘤患者5年生存率为60.5%。女性患者的1年、3年和5年总生存率分别为100%、91.7%（95%CI: 53.6%-98.8%）和73.3%（95%CI: 24.3%-93.4%）；男性患者的1年、3年总生存率以及中位生存期分别为96.2%（95%CI: 75.1%-99.8%）、53.0%（95%CI: 23.3%-82.7%）和38.4个月。肿瘤直径≤3.0 cm的患者1年、3年和5年总生存率分别为100%、79.8%（95%CI: 61.8%-97.8%）和60.5%（95%CI: 32.5%-88.4%）；肿瘤为3.1 cm-4.0 cm时，1年、3年总生存率以及中位生存期分别为83.3%（95%CI: 27.4%-97.5%）、31.3%（95%CI: 1.3%-73.3%）和27.8个月。Schoellnast等^[28]^回顾性分析了33例NSCLC患者手术放化疗后复发灶行RFA，单个肺转移灶，肿瘤平均直径为28 mm（10 mm-75 mm）。中位随访时间为24个月（1个月-98个月），中位PFS为8个月，中位生存期为21个月。Cheng等^[29]^回顾性分析了12例I期-III期放疗后局部复发的NSCLC患者的热消融（RFA 2例，MWA 10例）结果，有放疗手术禁忌，肿瘤平均直径为34 mm（17 mm-61 mm），中位随访时间为19个月（1个月-98个月），中位生存期为35个月。刘宝东等^[30]^回顾性分析了20例I期-III期NSCLC术后肺部转移复发灶的RFA结果，肿瘤平均直径为（3.9±2.0）cm（2 cm-8 cm）。平均随访19个月，中位PFS为25.0个月，中位生存期为27.0个月；1年生存率为92.9%，2年生存率为57%；肿瘤直径≤3 cm与>3 cm两组的中位生存期分别为34.0个月和27.0个月（P=0.158）。

#### 3.3.2 寡转移

2008年Lencioni等^[31]^开展了前瞻多中心单臂II期临床研究（RAPTURE study），研究对象为20例寡转移或寡复发性NSCLC患者（术后复发或者肺转移，肺内肿瘤少于3个，肿瘤直径≤3.5 cm，不适合手术或放化疗，肿瘤平均直径22 mm）行RFA。1年中位生存率为70%，2年中位生存率为48%；1年肿瘤特异性生存率为92%，2年肿瘤特异性生存率为73%。

Wei等^[32]^开展了随机对照III期临床研究，对IIIB期-IV期NSCLC患者（对既往未接受手术的初治患者的原发灶，或者既往接受过手术的患者的最大肺转移灶）行MWA，研究分为MWA+化疗组（n=148）和化疗组（n=145），肿瘤平均直径36 mm（10 mm-130 mm）。中位随访时间为13.1个月和12.4个月，中位PFS为10.3个月和4.9个月，中位生存期为未记录和12.6个月。

Li等^[33]^回顾性分析了2000年1月-2012年1月接受含铂双药化疗的220例晚期NSCLC患者中的49例（28例IIIB期，21例IV期）患者行RFA。入组标准是一线化疗部分缓解（partial response, PR）或疾病稳定（stable disease, SD）、肿瘤直径≤5 cm，肿瘤病灶数≤3个，距离纵隔或支气管血管≥1 cm，肿瘤平均直径29 mm（14 mm-50 mm）。中位随访时间为19个月（6个月-34个月），中位PFS为16周（95%CI：14.5周-17.5周），中位生存期为14个月。Wei等^[34]^回顾性分析了79例103枚寡转移灶101例次MWA治疗结果。中位PFS和中位生存期分别为14.0个月和47.8个月。女性、异时性疾病、手术治疗原发肿瘤部位、通过MWA完全消融（HR=0.024, P<0.001）是PFS的独立预后因素。44例患者（55.7%）有并发症，其中21例患者（29.6%）发生了严重并发症。

#### 3.3.3 寡进展

刘宝东等^[35]^回顾性分析了EGFR-TKIs治疗获得性耐药的28例NSCLC患者采用RFA的疗效。平均随访17.25个月，局部进展率为10.7%（3/28），平均局部进展时间为16.6个月。平均肿瘤无进展时间为（24.55±5.36）（95%CI：14.04-35.05）个月；生存期为（25.57±5.45）（95%CI：14.88-36.27）个月。Ni等^[36]^回顾性分析了54例EGFR（+）NSCLC患者EGFR-TKIs治疗后颅外寡进展的MWA治疗结果：MWA+EGFR-TKIs（MWA组n=28），系统化疗（化疗组n=26）。两组中位PFS1（EGFR-TKIs治疗开始到第一次进展）相似（12.6个月 vs 12.9个月，HR=0.63）。与化疗组相比，MWA组患者的PFS2（第一次进展后MWA或化疗到第二次进展）（中位数：8.8个月 vs 5.8个月，HR=0.357）显著延长，生存期（中位数：27.7个月 vs 20.0个月，HR=0.238）更好。多因素分析确定MWA是PFS2和生存期的预后良好因素。Ni等^[37]^回顾性分析了71例EGFR（+）NSCLC患者EGFR-TKIs治疗后80个颅外寡进展的热消融治疗结果：肿瘤病灶数≤3个，EGFR-TKIs治疗获得性耐药，颅外寡进展所有病灶行RFA/MWA，肿瘤平均直径33 mm（10 mm-105 mm）。中位PFS1（EGFR-TKIs治疗开始到局部进展）为11.8个月；热消融后的中位PFS2（首次局部进展到热消融后再次局部进展）为10.0个月，中位总生存期为26.4个月。PFS1和PFS2与生存期高度相关，而PFS1与PFS2不相关。低进展性病变的数量与PFS2显著且独立相关。

#### 3.3.4 疗效及影响因素

热消融的局部控制率在前瞻性研究^[32,33]^中为82%-88%，回顾性研究^[28,30]^中为55%-92%。热消融治疗寡转移性NSCLC患者的中位生存期为14个月-35个月，中位PFS为4个月-10.3个月；1年、3年、5年生存率分别为70%-100%、53%-72.9%和14%-55.7%。肿瘤直径<3 cm的患者热消融效果好^[29,30]^。

#### 3.3.5 安全性

热消融相关并发症轻微，呈自限性，常见并发症包括疼痛、无需治疗的胸腔积液和自限性肺内出血，且不会延长住院时间。热消融后仅有3.3%-38.9%（平均11%）的气胸患者需要放置胸腔闭式引流管，但通常1 d内可拔出。

#### 3.3.6 适应证

热消融最佳适应证是不能接受手术切除者，并且位于肺实质内的<3 cm N_0-1_周围型NSCLC患者，包括寡转移、寡复发和寡进展。

## 4 展望

近年来，随着麻醉技术的完善、手术技巧的提高和围手术期管理的进步，中晚期肺癌的诊治已经到了微创和精准的时代，特别是随着分子靶向药物治疗和免疫治疗的飞速发展，寡转移性NSCLC包括寡复发和寡进展的局部治疗或局部巩固治疗越来越受到重视，相关研究正在进行中，比如LONESTAR研究（免疫±局部巩固治疗）、NORTHSTAR研究[EGFR（+）靶向±局部巩固治疗]、BRIGHTSTAR研究[ALK（+）靶向±局部巩固治疗]、NRG-LU002（系统治疗±局部巩固治疗）等。相信，MDT管理模式下的肺癌肺部寡转移的近远期疗效会得到显著改善。
